# The pseudogene problem and RT-qPCR data normalization; *SYMPK*: a suitable reference gene for papillary thyroid carcinoma

**DOI:** 10.1038/s41598-020-75495-7

**Published:** 2020-10-27

**Authors:** Seyed-Morteza Javadirad, Mohammad Mokhtari, Ghazal Esfandiarpour, Mohsen Kolahdouzan

**Affiliations:** 1 Department of Cell and Molecular Biology and Microbiology, Faculty of Biological Science and Technology, University of Isfahan, ,; 2 Department of Surgery, School of Medicine, Isfahan University of Medical Sciences, ,

**Keywords:** Cancer genetics, Gene expression, Thyroid cancer

## Abstract

In RT-qPCR, accuracy requires multiple levels of standardization, but results could be obfuscated by human errors and technical limitations. Data normalization against suitable reference genes is critical, yet their observed expression can be confounded by pseudogenes. Eight reference genes were selected based on literature review and analysis of papillary thyroid carcinoma (PTC) microarray data. RNA extraction and cDNA synthesis were followed by RT-qPCR amplification in triplicate with exon-junction or intron-spanning primers. Several statistical analyses were applied using Microsoft Excel, NormFinder, and BestKeeper. In normal tissues, the least correlation of variation (CqCV%) and the lowest maximum fold change (MFC) were respectively recorded for *PYCR1* and *SYMPK*. In PTC tissues, *SYMPK* had the lowest CqCV% (5.16%) and MFC (1.17). According to NormFinder, the best reference combination was *SYMPK* and *ACTB* (stability value = 0.209). BestKeeper suggested *SYMPK* as the best reference in both normal (r = 0.969) and PTC tissues (r = 0.958). *SYMPK* is suggested as the best reference gene for overcoming the pseudogene problem in RT-qPCR data normalization, with a stability value of 0.319.

## Introduction

Differential gene expression analysis enables us to understand the patterns of complex gene networks and regulatory elements, thereby providing insight into biological processes and disease origins^1^. Reverse transcription quantitative polymerase chain reaction (RT-qPCR) is the “gold standard” and the most sensitive and accurate method for comprehensively quantifying gene expression^2^. However, owing to the context of the RT-qPCR technique and presumable human errors, several biases can obfuscate the results of these assays, leading to inaccurate data interpretations^1,3^.

In order to obtain the highest possible RT-qPCR data accuracy, multiple levels of standardization must be carried out^4^. After routine standardization of samples and adjustment of RNA concentration, the heart of RT-qPCR fine-tuning is in the use of suitable reference genes. Numerous lines of experiments have shown the inappropriateness of commonly-used reference genes, expression of which could fluctuate in different experimental and pathological conditions^5–11^. Additionally, heterogeneity of intratumor and interpatient expression profiles, which are common characteristics of papillary thyroid carcinoma (PTC), magnify the problem of expression fluctuation in reference genes^12–16^.

Finally, pseudogenes, naturally existing sequences with resemblance to fully-functional genes, are another factor that is not considered with painstaking care in mRNA expression analysis. Although non-operational due to deleterious mutations or retro-transposition, these replicates of working genes must also be added to the experimental budget because they can confound RT-qPCR assay results^17^. As expression analysis of true-functional genes is a daily practice of molecular biologists, not addressing bias from pseudogenes in RT-qPCR quantifications can lead to great headaches^18^. A recent bioinformatics exploration of the standard calibration genes Glyceraldehyde 3-phosphate dehydrogenase (*GAPDH*) and β-actin (*ACTB*) designated these two genes as unreliable because of contamination by their ample pseudogenes^19^. Consequently, pseudogenes must be considered more precisely in order to diminish the inaccuracy of RT-qPCR, which is otherwise a fundamentally accurate technique. The venerable technique of DNase treatment followed by enzyme deactivation is one solution, but unfortunately such handling would lead to RNA degradation^20,21^. A second possible solution is the usage of exon-junction (intron-spanning) primers; these have bidirectional benefits, as they not only eliminate genomic DNA but also heterogeneous nuclear RNA^22^. Finally, finding suitable reference genes with the lowest variation alongside an absence of pseudogenes is necessary to ensure the most accurate results.

In this study, we try to bring about a substantial improvement in the field of RT-qPCR data normalization by introducing Symplekin (*SYMPK*) as a stably expressed reference gene without pseudogenes, suitable for normalization in both PTC tumors and adjacent normal tissues (Tables 1 and 2).

**Table 1:** The information of the primers was listed.

Gene symbol (Genbank accession number)	The sequence of Forward and Reverse primers	Primers attachment information			Primer type	Product length (bp)	Annealing temperature (°C)
		Location of attachment (exon length, bp)	Exact nucleotide attachment on exon	Spanning Intron (length, bp)			
GAPDH (NM_001256799.3)	F:CCACTCCTCCACCTTTGACG	Exon 7 (413)	(344–363)	Intron 7–8 (104)	Intron spanning	107	58
	R:CCACCACCCTGTTGCTGTAG	Exon 8 (271)	(19–38)				
TBP (NM_003194.5)	F:GGTTTGCTGCGGTAATCA	Exon 5 (92)	2–19	Intron 5–6 (2602)	Exon junction	100	61
	R:TGTTCTTCACTCTTGGCTCCTGT	Exon 5 (92)	Exon 5 (79–92)				
		Exon 6 (168)	Exon 6 (1–9)				
SYMPK (NM_004819.3)	F:ACGGTGCTGAGGGTCATTGA	Exon 18 (160)	135–154	Intron 18–19 (1295)	Intron spanning, Exon junction	146	60
	R:GAGGGTGGGACTTTGTCTGTGA	Exon 19 (109)	Exon 19 (98–109)	Intron 19–20 (294)			
		Exon 20 (101)	Exon 20 (1–10)				
PYCR1 (NM_006907)	F:CTTCACAGCAGCAGGCGTC	Exon 1 (553) and	Exon 1 (540–553)	Intron 1–2 (554)	Intron spanning, Exon junction	122	61
		Exon 2 (71)	Exon2 (1–5)				
	R:TCTCCTTGTTGTGGGGTGTC	Exon 3 (180)	Exon 3 (18–37)	Intron 2–3 (606)			
ACTB (NM_001101.5)	F:CTGGAACGGTGAAGGTGACA	Exon 6 (744)	Exon 6 (291–310)	Not applicable	Not applicable	140	61
	R:AAGGGACTTCCTGTAACAACGC	Exon 6 (744)	Exon 6 (409–430)				
HPRT1 (NM_000194.3)	F:CGTCGTGATTAGTGATGATG	Exon 1 (174) and	Exon 1 (168–174)	Intron 1–2 (13,020)	Exon junction	123	60
		Exon 2 (107)	Exon 2 (1–13)				
	R:CGTTCAGTCCTGTCCATA	Exon 2 (107) and	Exon 2 (98–107)	Intron 2–3 (1715)			
		Exon 3 (184)	Exon 3 (1–9)				
B2M (NM_004048.3)	F:TGAGTATGCCTGCCGTGTGA	Exon 2 (279)	Exon 2 (221–240)	Intron 2–3 (627)	Intron spanning, Exon junction	97	61
	R:ATCTTCAAACCTCCATGATGCT	Exon 3 (28)	Exon 3 (17–28)	Intron 3–4 (1250)			
		Exon 4 (1271)	Exon 4 (1–10)				
GUSB (NM_000181.4)	F:CGCCCTGCCTATCTGTATTC	Exon 5 (188)	Exon 5 (162–181)	Intron 5–6 (943)	Intron spanning	91	60
	R:TCCCCACAGGGAGTGTGTAG	Exon 6 (153)	Exon 6 (45–64)				

A total of eight reference genes were selected and the detailed information of corresponding primers was listed. RefSeq accession numbers of the selected reference genes, forward (F) and reverse (R) primer sequences, exact primer attachment sites at their target genes, total length of the involved exons and spanned introns, PCR product length and PCR product Tm, are listed. Intron spanning primers: primer pairs attaching within two consequential exons. Exon junction primer: primer with spanning attachment to consequential exons.

*GAPDH* glyceraldehyde-3-phosphate dehydrogenase, *TBP* TATA-box binding protein, *SYMPK* symplekin, *PYCR1* pyrroline-5-carboxylate reductase 1, *ACTB* actin beta, *HPRT1* hypoxanthine phosphoribosyltransferase 1, *B2M* beta-2-microglobulin, *GUSB* glucuronidase beta.

**Table 2:** Pathological characteristics of the tissues were listed.

Parameter	PTC	Adjacent normal tissue	Total
Tissue number	10	10	20
**Histopathology**			
Classical PTC	6		
Follicular variant-PTC	4		
**Age**			
< 55 years old	8		
≥ 55	2		
**Tumor size (cm)**			
≤ 1	4		
1–2	6		
**Capsular invasion**			
Negative	9		
Positive	1		
**Lymph-vascular invasion**			
Negative	6		
Positive	4		
**Lymph node metastasis**			
Negative	6		
Positive	4		
**TNM stage**^**a**^			
T1bN0M0	3		
T1aN1aM0	2		
T1bN1aM0	1		
T1bN1bM0	1		
T1aNxMx	1		
T1aNxM0	1		
T3N0M0	1		

PTC; papillary thyroid carcinoma,

^a^TNM staging system is developed and is maintained by the Union for International Cancer Control (UICC), TNM stands for Tumour, Node, Metastasis.

## Results

### RNA quality and quantity

The mean A260/A280 ratios obtained for PTC tissues and their adjacent normal tissues were 1.96 ± 0.11 and 1.97 ± 0.06, respectively. The intensity of 28srRNA bands was approximately two-fold that of 18srRNA, which indicates integrity of the extracted mRNAs.

### Expression analysis of candidate reference genes

**Figure 1:**
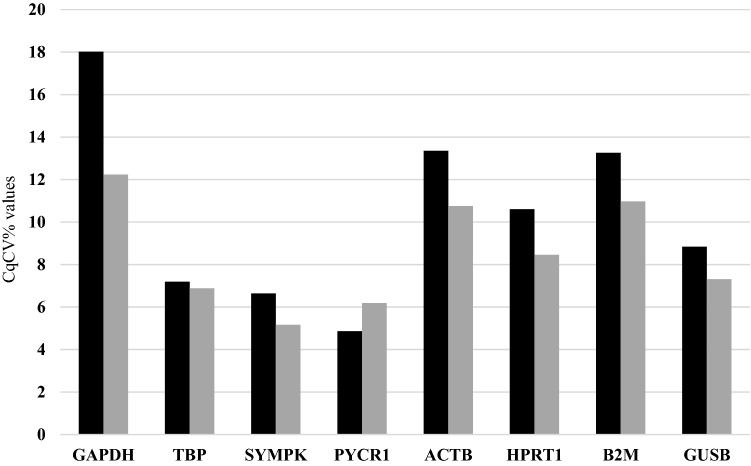
Basic statistical analysis representing CqCV% value of each reference gene. *PYCR1*, *SYMPK*, and *TBP*, respectively showed the lowest CqCV% values in normal tissues (black bars). The least CqCV% values were obtained for *SYMPK*, *PYCR1*, and *TBP*, respectively in PTC tissues (gray bars). The illustration confirmed pseudogene free-*SYMPK* with the lowest CqCV% value in PTC tissues.

Results from basic statistical analysis of the eight candidate reference genes in both normal and PTC tissues are shown in Table 3. In normal tissues, the least CqCV% values were recorded for *PYCR1* (4.86), *SYMPK* (6.64), and *TBP* (7.19), which genes also showed the lowest MFC values (1.16, 1.21, and 1.27, respectively). In PTC tissues, the least CqCV% values were obtained for *SYMPK* (5.16), *PYCR1* (6.19), and *TBP* (6.88), as were the lowest MFC values (1.17, 1.25, and 1.24, respectively). The representation of CqCV% was illustrated in Fig. 1.

**Table 3:** Basic statistics were presented.

Tissue	Gene	Mean Cq	SD	C_q_CV(%)	Minimum Cq	Maximum Cq	MFC
Normal	GAPDH	26.67	4.8	18.02	18.17	34.60	1.9
	TBP	30.64	2.20	7.19	26.45	33.83	1.27
	SYMPK	30.03	1.99	6.64	26.92	32.70	1.21
	PYCR1	33.99	1.65	4.86	31.15	36.21	1.16
	ACTB	26.06	3.48	13.35	21.78	31.75	1.45
	HPRT1	31.80	3.37	10.60	26.85	37.27	1.38
	B2M	24.82	3.29	13.26	20.39	29.77	1.46
	GUSB	32.73	2.89	8.84	28.82	38.15	1.32
PTC	GAPDH	24.73	3.02	12.23	19.34	29.63	1.53
	TBP^**a**^	31.01	2.13	6.88	28.01	34.93	1.24
	SYMPK^**a**^	29.88	1.54	5.16	27.22	32.04	1.17
	PYCR1^**a**^	33.66	2.08	6.19	28.84	36.10	1.25
	ACTB	25.13	2.70	10.75	21.21	29.51	1.39
	HPRT1	31.52	2.66	8.46	27.35	35.54	1.29
	B2M	24.80	2.72	10.97	20.81	30.45	1.46
	GUSB	32.65	2.38	7.31	28.69	36.27	1.26

^a^SYMPK, TBP, and PYCR1 were the best according to basic analysis using Microsoft Excel 2013 spreadsheet.

*Cq* cycle of quantification, *SD* standard deviation, *CqCV* Cq coefficient of variation, *MFC* maximum fold change.

### Identification of the most stable reference genes using NormFinder

**Figure 2:**
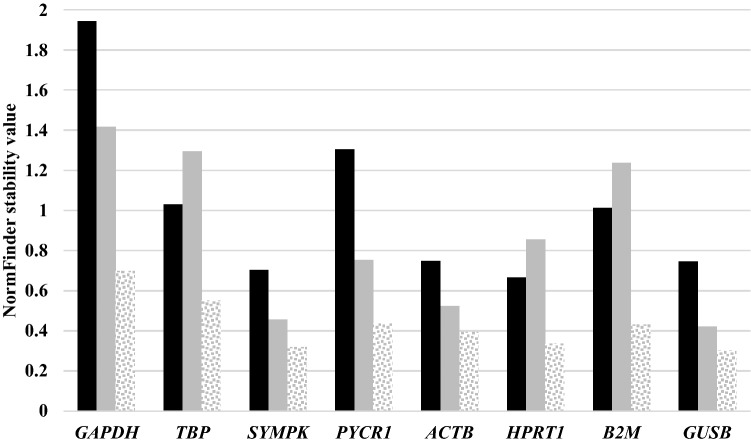
Intragroup and Intergroup Stability Value by NormFinder. *HPRT1*in normal tissues (black bar), and *GUSB* in PTC tissues (gray bars), were the most stable reference genes. *SYMPK* was the second most stable reference gene in both normal and PTC tissues. *SYMPK* with the narrowest margin, after *GUSB*, became a good candidate in Intergroup analysis (dotted gray).

NormFinder intragroup analysis was initially used to rank the candidate reference genes from most to the least stable (Table 4). In normal tissues, *HPRT1* was the most stable reference gene, followed by *SYMPK*, *GUSB*, *ACTB*, *B2M*, *TBP*, *PYCR1*, and finally *GAPDH* (black bar, Fig. 2). In PTC tissues, *GUSB* was the most stable reference gene, followed by *SYMPK*, *ACTB*, *PYCR1*, *HPRT1*, *B2M*, *TBP*, and finally *GAPDH* (gray bars, Fig. 2). Thus, NormFinder considered *HPRT1* and *GUSB* as the best reference genes for normal and PTC tissues respectively, while *SYMPK* was the second most stable reference gene in both tissue types.

**Table 4:** NormFinder analysis was presented.

Analysis type	Tissue	Candidated genes	Stability value
Intragroup	Normal	GAPDH	1.941
		TBP	1.030
		SYMPK	0.704
		PYCR1	1.305
		ACTB	0.747
		HPRT1	0.667
		B2M	1.013
		GUSB	0.745
	PTC	GAPDH	1.416
		TBP	1.295
		SYMPK	0.458
		PYCR1	0.754
		ACTB	0.524
		HPRT1	0.856
		B2M	1.237
		GUSB	0.421
Intergroup	Normal vs. PTC	GAPDH	0.698
		TBP	0.551
		SYMPK^a^	0.319
		PYCR1	0.436
		ACTB	0.395
		HPRT1^a^	0.337
		B2M	0.432
		GUSB^a^	0.301
	Best combination of two genes	SYMPK and ACTB	0.209

NormFinder analysis was used to rank the candidate reference genes. NormFinder considered *HPRT1* and *GUSB* as the best reference genes for normal and PTC tissues respectively, while *SYMPK* was the second most stable reference gene in both tissue types. Intergroup analysis indicated *SYMPK* and *ACTB*, with stability value of 0.209, as the best combination of reference genes.

^a^GUSB, SYMPK, and HPRT1 were the best according to the Normfinder algorithm.

Next, intergroup analysis was performed, checking reference gene performance in PTC tissues against normal tissues. In this analysis, NormFinder declared as the single best reference genes *GUSB*, *SYMPK*, and *HPRT1* (dotted gray, Fig. 2), and the best combination of reference genes as *SYMPK* and *ACTB*, with a stability value of 0.209 (Table 4).

### Identification of the most stable reference genes using BestKeeper

**Figure 3:**
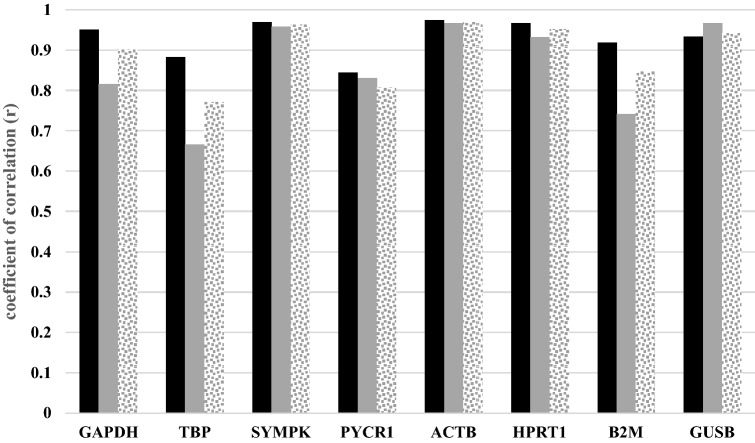
Intragroup and intergroup stability value by BestKeeper. Normal tissues (black bars), PTC tissues (gray bars), intergroup analysis (gray dotted bars).

According to BestKeeper intragroup analysis (Table 5), the most stable gene in normal tissues was *ACTB*, with r = 0.974. On the other hand, the most stable genes in PTC tissues were *ACTB* and *GUSB*, which tied with r = 0.966. In normal tissues, the second and third most stable genes were *SYMPK* and *HPRT1*, with r = 0.969 and r = 0.966, respectively. The same ranking was observed in PTC tissues, with *SYMPK* (r = 0.958) and *HPRT1* (r = 0.931) having the second and third highest similarity to the BestKeeper index. Finally, PTC and normal intergroup analysis using BestKeeper ranked the candidate genes from most to least stable as follows: *ACTB*, *SYMPK*, *HPRT1*, *GUSB*, *GAPDH*, *B2M*, *PYCR1*, and *TBP* (Fig. 3).

**Table 5:** BestKeeper analysis was presented.

Analysis type	Tissue	Symbol gene	Coefficient of correlation (r)
Intragroup	Normal	GAPDH	0.950
		TBP	0.883
		SYMPK	0.969
		PYCR1	0.844
		ACTB	0.974
		HPRT1	0.966
		B2M	0.918
		GUSB	0.933
	PTC	GAPDH	0.816
		TBP	0.666
		SYMPK	0.958
		PYCR1	0.831
		ACTB	0.966
		HPRT1	0.931
		B2M	0.741
		GUSB	0.966
Intergroup	Normal vs. PTC	GAPDH	0.903
		TBP	0.771
		SYMPK^a^	0.963
		PYCR1	0.807
		ACTB^a^	0.969
		HPRT1^a^	0.951
		B2M	0.847
		GUSB	0.941

BestKeeper intragroup analysis showed *ACTB* and *ACTB*/ *GUSB* as the most stable genes in normal and PTC tissues respectively. The second most stable gene was *SYMPK* according to the BestKeeper index. Finally, PTC and normal intergroup analysis ranked the candidate genes from most to least stable as follows: *ACTB*, *SYMPK*, *HPRT1*, *GUSB*, *GAPDH*, *B2M*, *PYCR1*, and *TBP*.

^a^ACTB, SYMPK, and HPRT1 were the best according to the BestKeeper algorithm.

## Discussion

When performing qPCR normalization, a reference gene could be considered reliable if it is expressed consistently throughout different pathological statuses^5^. While truly constant expression might only be seen in fairy tales, a lack of confounding influence such as from pseudogenes can be achieved^5^. Omitting pseudogene signal could bring more stability to the observed expression of reference genes, hence our current approach of finding and validating genes with minimal expression variation and that lack pseudogenes^19,23^.

Different statistical parameters could affect the interpretation of reference gene stability. The first factor that came to our attention was CqCV%, as it is more comprehensive than mean and SD. It must be mentioned that CqCV% or distribution frequency is a statistical factor that measures the dispersion of the data and the repeatability and precision of the experiment^24^. In this study, *SYMPK* and *PYCR1* showed the lowest CqCV% in both tissues, meaning that these two genes had the lowest dispersion around the mean value. MFC is another basic statistical factor that indicates the dispersion, the variation, and the difference between minimum and maximum Cq. In the same manner, *SYMPK* and *PYCR1* showed the lowest MFC in both tissues.

According to the NormFinder algorithm, the best single reference gene was *GUSB* (stability value = 0.301); however, *ACTB* and *SYMPK* were suggested as the best combination, with a stability value of 0.209. Interestingly, despite fundamental differences in the analytical software, the BestKeeper algorithm suggested exactly the same two genes as the most stable. This aligns well with the basic statistical analysis, in which *SYMPK* also had the best MFC value and CqCV%, and thus the aforementioned predictions from microarray data were in accordance with our labwork.

To date, very few studies have investigated reference genes in thyroid cell lines and human tissues. A previous study of six candidate reference genes (*ACTB*, *B2M*, *HPRT1*, *GAPDH*, *SDHA*, and *YWHAZ*) in seven goiter and seven normal tissues met with failure when intragroup analysis was done^25^. However, they declared *SDHA* and *ACTB* as the most stable reference genes based on NormFinder intergroup analysis^25^. In a same manner, this study found *ACTB* alone was not a well-qualified reference gene for intragroup analysis, but *SYMPK*/*ACTB* in combination had advantages in terms of basic statistics. Unfortunately, *ACTB* suffers from considerable pseudogene bias, and thus its reliability as a reference gene is dubious. Another study of 45 tissue samples from Iranian patients (15 PTC, 15 paired normal and 15 goiter tissues) checked twelve genes (*GAPDH*, *ACTB*, *HPRT1*, *TBP*, *B2M*, *PPIA*, *18SrRNA*, *HMBS*, *GUSB*, *PGK1*, *RPLP0*, and *PGM1*)^26^. They declared consistency between the different algorithms of NormFinder, BestKeeper, and GeNorm because all three suggested *GUSB* and *HPRT1* as the most stably expressed genes in all thyroid tumours. In line with this study, we found *GUSB* to be a stable reference gene, but *GUSB* also has numerous pseudogenes that can obfuscate RT-qPCR data normalization. *HPRT1* was not considered a suitable reference gene in our study as it was only the third most stable reference gene according to both NormFinder and BestKeeper.

Considering the limitations described above, the ideal combination of *SYMPK*/*ACTB*, despite its stability value of 0.209, could not in fact be appropriate for RT-qPCR normalization. Pseudogene contamination of *ACTB* and also *GUSB* put these two genes out of the running^19,27–29^. As pseudogene can cause falsely lower Cq values due to undesirable mRNA amplification, therefore *ACTB* cannot be helpful beside *SYMPK*. To avoid the problem of pseudogenes, the next most stable gene—*SYMPK*, with a stability value of 0.319—is the most suitable reference.

According to our knowledge, *SYMPK* was suggested as the second suitable reference gene only in one previous study; albeit after *CCSER2* gene^30^. CCSER2\*SYMPK* inauguration was launched after transcriptome and microarray data analysis in breast cancer cell lines and tissues. Unfortunately we found *CCSER2* with a pseudogene (*LOC100127962*) sufficiently enough to be ruled out of this study. As a final point, *SYMPK* with the lack of pseudogenes and being presented only as a single isoform within cells showed mastery over the other competitor and it was selected in this study.

## Conclusion

Synchronous results of BestKeeper and NormFinder suggested the combination of *SYMPK* and *ACTB* for RT-qPCR data normalization, but confounding from *ACTB* pseudogenes takes it out of the running and leaves SYMPK as the champion.

## Subjects and methods

### Reference gene selection

A total of eight reference genes were selected, with six based on literature review: *SYMPK*^30^, Beta-2-microglobulin (*B2M*)^25^, *GAPDH*^26,31,32^, TATA-box binding protein (*TBP*)^26,33^, *ACTB*^26,34^, and Hypoxanthine phosphoribosyltransferase 1 (*HPRT1*)^26,34^. The other two candidate reference genes were Pyrroline-5-carboxylate reductase 1 (*PYCR1*), which promotes cell proliferation in different human neoplasms but surprisingly does not fluctuate in PTC tumors (unpublished data), and β-glucuronidase (*GUSB*), selected based on a recently published study^26^.

### Reference gene validation using microarray data

Our selected reference genes were validated using the GSE3678 microarray dataset, which contains seven PTC samples and seven adjacent normal tissues. The dataset was checked for log scale and the quality of the data was assessed. Quantile normalization of the dataset was checked and principal component analysis (PCA) was performed (Supplementary Fig. 1). Finally, a list of genes with minimal variation was extracted and the presence of our selected reference genes in that list was confirmed. The microarray dataset was analyzed with an R program (script attached).

### Exon-junction primer design

Exon-junction primer design was undertaken in order to eliminate pseudo-amplification of genomic DNA and\or heterogeneous nuclear RNA^35^. Seven pairs of exon-junction primers were designed using Beacon Designer 8.1 (Premier Biosoft International, Palo Alto, CA, USA). To do this, the exact positions of introns were extracted from the Ensemble Genome Browser, and the exact nucleotide sequences of exons were imported into Beacon Designer 8.1 separately for each of our seven mRNAs. At the final step, the software was asked to design primers that span selected exon(s) in order to specifically amplify mature mRNAs of the desired genes. Primers that did not pass the exon-spanning requirement were rejected and not included in the study. For the eighth candidate gene, *GUSB*, primer sequences were taken directly from a recently published study^26^. Secondary structures of the primers were re-checked using Oligo7, and to insure the selective amplification of mature mRNAs, specificity was confirmed using NCBI-primer BLAST. Melting temperatures for all pairs of primers were validated using gradient PCR (Sinaclon Bioscience, Tehran, Iran). The complete details of the designed primers, including exon-junction information, are listed in Table 1.

### Tissue acquisition

A written informed consent was obtained before tissue acquisition. The study was approved by the Iran National Committee for Ethics in Biomedical Research Review Board and the Ethics Committee of Research Institute for Endocrine Sciences, Isfahan University of Medical Sciences, Isfahan, Iran (IR.UI.REC.1398.058). PTC and adjacent normal tissues were obtained from patients undergoing thyroidectomy at the Alzahra and Sina hospitals, Isfahan province, Iran. Tissues were immediately submerged in adequate RNAlater RNA Stabilization Reagent (Qiagene, Hilden, Germany) and incubated at 4 °C for 24 h according to the manufacturer’s instruction. If needed, samples were stored at − 80 °C. According to the postoperative pathological and histological examinations, of the 20 thyroid tissue samples, ten were PTC and ten were adjacent normal thyroid tissues, each with the approximate size of 0.5 cm. Histopathological examinations were done by either the operating hospital or a third-party laboratory. The staging of the tumors was determined by specialized pathologists according to the 7^th^ edition of the American Joint Committee on Cancer Tumor-Node-Metastasis (TNM) staging system. However, because of the restricted number of other thyroid neoplasm tissues in Iran, the data presented here are restricted to PTC tissues. We had only one anaplastic thyroid cancer sample, which was not included in the study. Patients’ information and tumour pathological characteristics were presented in Table 2.

### RNA extraction and quantification

Total RNA was extracted from RNAlater-treated tissues using a one-step RNA extraction reagent (BIOBASIC, Canada), according to the manufacturer’s instructions. The concentration of the isolated RNA was measured using a NanoDrop oneC spectrophotometer (ThermoScientific, Waltham, MA, USA), and quality was determined using the A260/A280 and A260/A230 ratios. The integrity of extracted RNA was confirmed by 1.0% agarose gel electrophoresis (Invitrogen).

### Complementary DNA (cDNA) synthesis

DNase I (ThermoScientific, Germany) was applied to eliminate residual genomic DNA according to the manufacturer’s instruction. The ThermoScientific RevertAid Reverse Transcriptase kit was used to reverse transcribe 1 μg of total RNA according to the manufacturer's instruction. The reaction was done in a total volume of 20 μL.

### Reverse transcription quantitative polymerase chain reaction (RT-qPCR)

RT-qPCR reactions were performed in a Bio-Rad Chromo4 machine using SYBR Green RealQ Plus 2 × Master Mix (Ampliqon, Odense, Denmark). The RT-qPCR reaction was as follows: one cycle of enzyme activation and initial denaturation at 95 °C for 15 min, then 40 cycles of 95 °C for 30 s, specific primer annealing temperature for 30 s, and 72 °C for 30 s. A plate reading was carried out after each cycle. All RT-qPCR reactions were run in triplicate, and a non-template control (NTC) was used for each run.

### Melt curve analysis

The specificity of the RT-qPCR was evaluated using melt curve analysis through the gradual increase of temperature with a transition rate of 1 °C (from 55 to 95 °C). After each temperature increase, a plate reading was performed on the green channel. The derivative of fluorescence change over temperature (y axis) was plotted against the temperature (°C, x axis).

### Statistical analysis

Microsoft Excel 2013 was used to calculate the mean C_q_, standard deviation (SD), correlation of variation (C_q_CV%, C_q_CV% = SD/mean × 100%), minimum C_q_, maximum C_q_, and maximum fold change (MFC) (Table 1). MFC was calculated by dividing the maximum Cq by minimum Cq; this value is an estimate that represents the distribution of the Cq, with a lower MFC value indicating a narrower distribution of a reference gene Cq^25^. NormFinder^36^ and BestKeeper^24^ were used to validate the appropriateness of reference genes. NormFinder ranks reference genes based on their stability value, with lower stability value indicating a more stable reference gene^36^. BestKeeper creates its reference gene index using pair-wise correlations based on the average C_q_ values, SD, and CV^24^. This algorithm considers the gene with the highest Pearson’s correlation coefficient (r) as the most stable reference gene.

### Ethical approval

The study was approved by the Iran National Committee for Ethics in Biomedical Research Review Board and the Ethics Committee of Research, Institute for Endocrine Sciences, Isfahan University of Medical Sciences, Isfahan, Iran (IR.UI.REC.1398.058). All experiments involving participants were performed in accordance with the seventh edition of the Helsinki declaration and the research protocol was first submitted to the Iran National Committee for Ethics in Biomedical Research Review Board and the Ethics Committee of Research, Institute for Endocrine Sciences, Isfahan University of Medical Sciences. Informed consent, including the aims, methods, and sources of funding, institutional affiliations of the researchers, the anticipated benefits and potential risks of the study, and post-study provisions, was obtained for each participant. We also informed each participant of their right to refuse.

## Supplementary information

Supplementary Information 1.

Supplementary Information 2.

## Data Availability

The dataset analyzed during the current study is available in the NCBI-Gene Expression Omnibus repository, [https://www.ncbi.nlm.nih.gov/sites/GDSbrowser?acc=GDS1732].
