# Supplementary material for: The pseudogene problem and RT-qPCR data normalization; SYMPK: a suitable reference gene for papillary thyroid carcinoma
Source: Sci Rep. 2020 Oct 27;10:18408. doi: 10.1038/s41598-020-75495-7 (PMC7592052; doi:10.1038/s41598-020-75495-7)

Supplementary

Original paper

The pseudogene problem and RT-qPCR data normalization; *SYMPK*: a suitable reference gene for papillary thyroid carcinoma

Seyed-Morteza Javadirad[1*], Mohammad Mokhtari[1a], Ghazal Esfandiarpour [1a], Mohsen kolahdouzan[2]

Supplementary

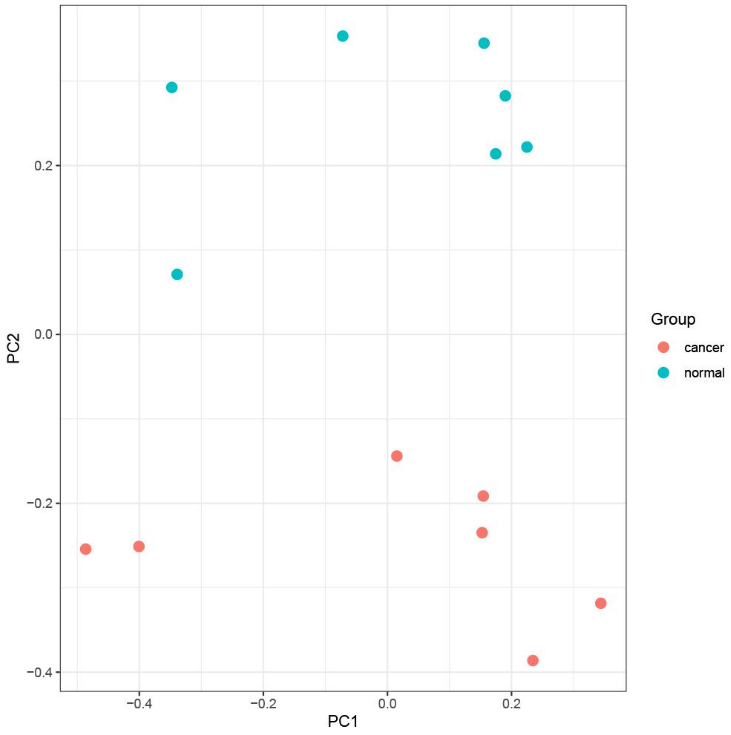

Figure 1: PCA plot representing PTC tissues (red dots) and normal tissues (blue dots). Separate grouping of PTC and normal tissues, indicates good quality of microarray dataset GSE3678.

TNM guideline used in the study:

One tool that doctors use to describe the stage is the TNM system. Doctors use the results from diagnostic tests and scans to answer these questions:

Tumor (T): How large is the primary tumor? Where is it located?

Node (N): Has the tumor spread to the lymph nodes? If so, where and how many?

Metastasis (M): Has the cancer spread to other parts of the body? If so, where and how much?

The results are combined to determine the stage of cancer for each person. For thyroid cancer, there are 5 stages: stage 0 (zero) and stages I through IV (1 through 4). The stage provides a common way of describing the cancer, so doctors can work together to plan the best treatments.

In addition to the TNM system, papillary and follicular thyroid cancers are also staged based on the age of the patient.

Staging can be clinical or pathological. Clinical staging is based on the results of tests done before surgery, which may include physical examinations and imaging tests. Pathological staging is based on what is found during surgery, including a biopsy. In general, pathological staging provides the most information to determine a patient's prognosis.

Here are more details on each part of the TNM system for thyroid cancer:

Tumor (T)

Using the TNM system, the letter "T" plus a letter or number (0 to 4) is used to describe the size and location of the tumor. Tumor size is measured in centimeters (cm). A centimeter is roughly equal to the width of a standard pen or pencil.

Stages may also be divided into smaller groups that help describe the tumor in even more detail. When describing "T" in thyroid cancer, doctors may subdivide the general categories by adding the letter "s" to indicate a solitary (single) tumor or "m" to indicate multifocal (more than 1) tumors. Specific tumor stage information is listed below.

TX: The primary tumor cannot be evaluated.

T0 (T plus zero): There is no evidence of a tumor.

T1: The tumor is 2 centimeters (cm) or smaller and limited to the thyroid.

T1a: The tumor is 1 cm or smaller.

T1b: The tumor is larger than 1 cm but less than 2 cm.

T2: The tumor is larger than 2 cm but smaller than 4 cm and is limited to the thyroid.

T3: The tumor is larger than 4 cm, but the tumor does not extend beyond the thyroid gland.

T4: The tumor is any size and has extended beyond the thyroid.

T4a: The tumor has spread beyond the thyroid to nearby soft tissues, the larynx, trachea, esophagus, or recurrent laryngeal nerve.

T4b: The tumor has spread beyond the regions in T4a (above).

Node (N)

The “N” in the TNM staging system stands for lymph nodes. Careful evaluation of lymph nodes is an important part of staging thyroid cancer. There are many regional lymph nodes located in the head and neck area. Lymph nodes in other parts of the body are called distant lymph nodes.

NX: The regional lymph nodes cannot be evaluated.

N0 (N plus zero): There is no evidence of cancer in the regional lymph nodes.

N1: Cancer has spread to the lymph nodes.

N1a: Cancer has spread to the lymph nodes around the thyroid (called the central compartment; the pretracheal, paratracheal, and prelaryngeal lymph nodes).

N1b: Cancer has spread beyond the central compartment, including unilateral cervical (lymph nodes on 1 side of the neck), bilateral cervical (lymph nodes on both sides of the neck), contralateral cervical (the opposite side of the tumor), or mediastinal (the chest) lymph nodes.

Metastasis (M)

The “M” in the TNM system describes whether cancer has spread to other parts of the body, called distant metastasis.

MX: Distant metastasis cannot be evaluated.

M0 (M plus zero): Cancer has not spread to other parts of the body.

M1: Cancer has spread to other parts of the body.

Cancer stage grouping

Doctors assign the stage of the cancer by combining the T, N, and M classifications. For thyroid cancer, this staging system differs by tumor type. For papillary or follicular thyroid cancer, staging also depends on the age of the patient.

Papillary or follicular thyroid cancer in a person younger than 55

Stage I: This stage describes a tumor (any T) with or without spread to lymph nodes (any N) and no distant metastasis (M0).

Stage II: This stage describes a tumor (any T) with any metastasis (M1) regardless of whether it has spread to the lymph nodes (any N).

Papillary or follicular thyroid cancer in a person 55 and older

Stage I: This stage describes any small tumor (T1) with no spread to lymph nodes (N0) and no metastasis (M0).

Stage II: This stage describes a larger, noninvasive tumor (T2) with no spread to lymph nodes (N0) and no metastasis (M0).

Stage III: This stage describes a tumor larger than 4 cm but still contained in the thyroid (T3) with no spread to lymph nodes (N0) and no metastasis (M0). Or, any localized tumor (T1, T2, or T3) with spread to the central compartment of lymph nodes (N1a) but no distant spread (M0).

Stage IVA: This stage describes a tumor that has spread to nearby structures (T4a), regardless of whether it has spread to the lymph nodes (any N), but it has not spread to distant places (M0). Or, this describes a localized tumor (T1, T2, or T3) with lymph node spread beyond the central compartment (N1b) but no distant spread (M0).

Stage IVB: This stage describes a tumor that has spread beyond nearby structures (T4b), regardless of spread to lymph nodes (any N), but no distant spread (M0).

Stage IVC: This stage describes all tumors (any T, any N) when there is evidence of metastasis (M1).

Pat-9116

MACROSCOPIC DESCRIPTION

Total thyroidectomy:

One lobe measure 4*2.5*2 cm. with well defined solid mass gray to white (1.2 cm .in diameter).

The other lobe measure 5*2.5*2 cm. with 4 well-defined solid masses gray to white (1.5, 1.3, 1 and 0.6 cm in diameter).

DX: THYROIDECTOMY :

One Lobe (probably left) : -Tumor Type: Papillary Thyroid Carcinoma, Classic & oncocytes Type

-Tumor Size: 1.2 cm

-Tumor Mitosis: Low

-Tumor Necrosis: Absent

-Nuclear Atypia: Present, mild

-Tumor Dedifferentiation: Absent

-Encapsulation: Absent

-Capsular Invasion: Absent

-Closest Distance to Resected Margin: 0.1 cm

-Vascular invasion: Absent

-Perineurial Invasion: Absent

-Extrathyroid Extension: Absent

-Surgical Margin : Free of tumor

-Additional Pathologic Findings: Normal thyroid tissue

Pat-9116

The other Lobe (probably right lobe): -Tumor Type: Papillary Thyroid Carcinoma & microcarcinomas , all are Classic Type

-Tumor Size: 1.5 , 1.3 , 1 , 0.6 cm

-Tumor Mitosis: Low

-Tumor Necrosis: Absent

-Nuclear Atypia: Present, mild

-Tumor Dedifferentiation: Absent

-Encapsulation: Absent

-Capsular Invasion: Absent

-Vascular invasion: Absent

-Perineurial invasion: Absent

-Extrathyroid Extension: Absent

-Surgical Margin: Thyroid capsule is involved by tumor

-Tumor Multicentricity: Present (right & left)

-Additional Pathologic Findings: Normal thyroid tissue

Pathologic stage :T3 NO M0:stage I

Surgical Pathology Report

➢ ***Specimen Origin:***

Total thyroidectomy with left & right lateral lymph nodes dissection

➢ ***Macroscopic Evaluation***

Received specimen consisted of three formalin filled containers:

Thyroid included total thyroidectomy,Rt.lobe dimension: 3x2x1cm,color: grayish-brown, cut surface contained one mass with diameter:0.5 cm.

Lt.lobe: dimension: 3.5x2.5x2 cm,color: grayish-brown, cut surface contained one mass with diameter:1.5 cm.

Right lateral lymph nodes included one fragment measured:2x0.9x0.4 cm. Totally 1 lymph node was isolated.

Left lateral lymph nodes included two fragments measured:3x2.5x1 cm. Totally 3 lymph node was isolated.

➢ ***Microscopic Evaluation:***

Sections showed a neoplastic proliferation of epithelial cells forming follicles a few papilla containing fibrovascular core. These structures lined by single layered epithelial cells containing ground glass nuclei and intranuclear groove.Nuclear overlapping is also seen.

Right lateral lymph node (Totally 1) was free.

Left lateral lymph nodes (Totally 3) were free.

<u>Total thyroidectomy with left & right lymph nodes dissection:</u>

1.Papillary thyroid carcinoma of Lt.thyroid lobe with diameter:1.5cm

Papillary thyroid carcinoma of Rt.thyroid lobe with diameter:0.5 cm

Thyroid capsule was intact

2.Right lateral lymph node (Totally 1) was free.

3.Left lateral lymph nodes (Totally 3) were free.

Pathologist

آزمایشگاه تشخیص طبی و پاتولوژی دکتر شیرنشان
خیابان شمس آبادی-روبروی بیمارستان سینا-ساختمان فارابی-۳۳۲۲۴۵۰۳۰۲۱

P98_69949

MACROSCOPIC DESCRIPTION:

The specimens consists of two containers:

1- Total Thyroidectomy: *Consists of thyroid weigh 15g, right lobe measures 4x1.5x1cm and the left lobe measures 4.2x2x1.2 cm , with brown color . In cut section has brown and light creamy color. It reveals a whitish area measures 0.8cm in diameter, next to superior pole of right lobe. In cut section in the left lobe reveals a whitsh nodule measures 1.5cm in diameter. Calcified area is seen. There is a separated piece of fat tissue measures 4x2.5x0.5cm, contains 7 lymph nodes, the greatest measures 0.5cm in diameter.*

2- Left Neck Lymphadenectomy: *Consists of 2 lymph nodes, the greater measures 3.5x1x0.7cm, with a follicular appearance.*

MICROSCOPIC DESCRIPTION:

1- Total Thyroidectomy: *In left lobe sections show foci of f ollicular variant of papillary carcinoma, with no papillae, but nuclear enlargement, ovarlapping, nuclear membrane irregularities & margination of chromatin to nuclear membrane are seen. Psammoma bodies and abortive papillae are seen.*

2- Left Neck Lymphadenectomy: *Both 2 lymph nodes are involved by metatatic papillary carcinoma of thyroid.*

Dx: 1- TOTAL THYROIDECTOMY:

- FOLLICULAR VARIANT OF PAPILLARY THYROID CARCINOMA OF LEFT THYROID LOBE.

- ***Tumor size: 1.5cm***
- ***Vascular invasion: - Absent.***
- ***Mitotic rate: 0-1 / 10 HPF***
- ***Lymphatic invasion: - Absent.***
- ***Microscopic extrathyroid tumor extension: - Absent.***
- ***Microscopic resection margins: - Negative.***
- ***Regional lymph nodes are not involved (0/7).***

2- LEFT NECK LYMPHADENECTOMY:

- METASTATIC LYMPHADENOPATHY (2/2).

With The Best Regards: K.Shirneshn MD./F.P.C.P.

آزمایشگاه تشخیص طبی و پاتولوژی دکتر شیرنشان
خیابان شمس آبادی-روبروی بیمارستان سینا-ساختمان فارابی-۰۳۱۳۲۲۳۶۵۰۳۰ p98_70070

MACROSCOPIC DESCRIPTION:

The specimens consists of 3 containers:

*1- **Total Thyroidectomy:** Consists of thyroid weigh 33g, with brown color and nodular appearance, right lobe measures 4.5x3x2cm, with nodular appearance in cut surface. It reveals a whitish nodule measures 0.6 cm in diameter , next to superior pole of right lobe . The left lobe has also nodular appearance.*

*2- **Right Neck Lymphadenectomy:** Consists of 2 pieces, the greater measures 2.8x1.5x1.5cm, with yellow color, contains 7 lymph nodes, the greatest measures 1cm in diameter, with a whitish color*

*3- **Central Neck Lymphadenectomy:** Consists of 3 pieces, the greatest measures 1.9x1.1x1cm, with a whitish color and firm consistency in cut section , contains 5 lymph nodes , the greatest measures 1cm in diameter, with a whitish color.*

MICROSCOPIC DESCRIPTION:

*1- **Total Thyroidectomy:** In right lobe sections show foci of follicular variant of papillary carcinoma, with no papillae, but nuclear enlargement, ovarlapping, nuclear membrane irregularities & margination of chromatin to nuclear membrane are seen. Psammoma bodies and abortive papillae are seen.*

*2- **Right Neck Lymphadenectomy:** 4 lymph nodes out of 7 lymph nodes are involved by metatatic papillary carcinoma of thyroid (4/7).*

*3- **Central Neck Lymphadenectomy:** 4 lymph nodes out of 5 lymph nodes are involved by metatatic papillary carcinoma of thyroid (4/5).*

Dx: 1- TOTAL THYROIDECTOMY:

- FOLLICULAR VARIANT OF PAPILLARY THYROID CARCINOMA OF RIGHT THYROID LOBE.

- *Tumor size: 0.6cm*
- ***Mitotic rate: 0-1 / 10 HPF***
- ***Vascular invasion: - Absent.***
- ***Lymphatic invasion: - Present.***
- ***Microscopic extrathyroid tumor extension: - Absent.***
- ***Microscopic resection margins: - Negative.***

2- RIGHT NECK LYMPHADENECTOMY:

- METASTATIC PAPILLARY CARCINOMA (4/7).

3- CENTRAL NECK LYMPHADENECTOMY:

- METASTATIC PAPILLARY CARCINOMA (4/5).

With The Best Regards: K.Shirneshn MD. AP.CP

دکتر کیوان شیرنشان
بورد تخصصی آناتومیکال و کلینیکال پاتولوژی

آزمایشگاه تشخیص طبی و پاتولوژی دکتر شیرنشان
P98_70072 ۱۳۲۲۳۶۵۰۳۰ [illegible]

MACROSCOPIC DESCRIPTION:

The specimens consists of two containers:

***1- Total Thyroidectomy:** Consists of thyroid weigh 16g , right lobe measures 5x2x1.5 cm. It reveals a whitish nodule measures 1cm in diameter, in the center of the right lobe. The left lobe measures 4.5x2x1.7cm.*

***2- Central Neck Lymphadenectomy:** Consists of 4 lymph nodes, the greatest measures 0.6cm in diameter, with brown-gray color.*

MICROSCOPIC DESCRIPTION:

***1- Total Thyroidectomy:** In right lobe sections show foci of follicular variant of papillary carcinoma, with no papillae, but nuclear enlargement, ovarlapping, nuclear membrane irregularities & margination of chromatin to nuclear membrane are seen. Psammoma bodies and abortive papillae are seen. Stroma is fibrotic.*

***2- Central Neck Lymphadenectomy:** All of 4 lymph nodes are involved by metatatic papillary carcinoma of thyroid (4/4).*

Dx: 1- TOTAL THYROIDECTOMY:

- FOLLICULAR VARIANT OF PAPILLARY THYROID CARCINOMA OF RIGHT THYROID LOBE.

- *Tumor size: 1cm*
- *Mitotic rate: 0-1 / 10 HPF*
- *Lymphatic invasion: - Present.*
- *Vascular invasion: - Absent.*
- *Microscopic extrathyroid tumor extension: - Absent.*
- *Microscopic resection margins: - Negative.*

2- CENTRAL NECK LYMPHADENECTOMY:

- METASTATIC PAPILLARY CARCINOMA (4/4).

With The Best Regards: K.Shirneshn M.D. A.P.C.P

S98-235

واحد آزمایشگاه پاتولوژی

سطح ۴-آسیب شناسی تشریحی بررسی ظاهری بافت و ریزبینی میکروسکوپی(میکروسکوپی)شامل:تیروئید توتال/لوب

EXE.Time 1398/03/11 16:05 Result.Time 1398/03/21 16:36 Print time

Macroscopic

نمونه ارسالی شامل سه ظرف می باشد:

ظرف اول با برچسب تیروئید به وزن ۳۵ گرم می باشد. لوب راست به ابعاد ۵×۳/۵×۱ سانتیمتر، ناحیه ایسم به ابعاد ۲×۳×۰/۵ سانتیمتر و لوب چپ به ابعاد ۵×۳/۵×۱/۵ سانتیمتر است .در برش های متوالی از لوب راست و ایسم نکته پاتولوژیک دیده نشد.

در برش های متوالی از لوب چپ توده سفید رنگ با حدود.د مشخص با حداکثر قطر ۱/۳ سانتیمتر رویت شد.

ظرف دوم :غدد لنفاوی سمت راست شامل چندین قطعه بافت کرم خاکستری رنگ با قوام نرم به ابعاد ۶×۳/۵×۱/۵ سانتیمتر است .

در برش چند عدد کیست حاوی مایع غلیظ قهوه ای رنگ و سطح مقطع چند لنف نود رویت شد .

ظرف سوم :غدد لنفاوی سمت چپ شامل چندین قطعه بافت کیستیک حاوی مواد چسبناک سبز رنگ و چند عدد لنف نود می باشد.

S98-2235

Microscopic

-Procedure:*Total thyroidectomy*

Lymph node sampling:Lateral neck dissection ; right and left

Specimen integrity:Intact

-Tumor Size: Right lobe:5*3.5*1 cm

Left lobe:5*3.5*1.5 cm

Isthmus:2*3*0.5 cm

Specimen weights:35 gr

Tumor focality :Multifocal

Tumor laterality:Right and left lobe and isthmus

Tumor size:Greatest dimension:1.3 cm

-Histologic Type:Papillary carcinoma , classic

Tumor capsular invasion:Not identified

Angioinvasion:Absent

-Lymph-Vascular Invasion:Present

-Perineural Invasion:Not identified

-Extrathyroidal extension:Not identified

-additional finding:one parathyroid gland is seen

-Pathologic Staging:PT1b:Tumor more than 1 cm but not more than 4 cm limited to thyroid

PN1b:Metastasis to bilateral cervical

-Number of Lymph Nodes Examined:29

-Number of Lymph Nodes Involved:14

-Pathologic staging:Stage I (21 years old)

anyT anyN Mo age <55Y=stage I

آزمایشگاه تشخیص طبی و پاتولوژی دکتر شیرنشان
[illegible] ۰۳۱۳۲۲۳۶۵۰۳۰

MACROSCOPIC DESCRIPTION:

The specimen consist of two pieces totally weigh 11g. The first piece measures 4.5x2x1.3cm and the other piece measures 4.5x2x1.3cm. It reveals a nodule measures 1.1cm in diameter, with whitish color in the first piece. The second piece has a normal appearance in cut section.

MICROSCOPIC DESCRIPTION:

In one lobe sections show numeorus papillary structures which are lined by a single layer of tall cellsand abundant acidophilic cytoplasm. These cells have ground glass nuclei with a enlarged size & overlapping quality. Nuclear groove are also seen.

Dx: TOTAL THYROIDECTOMY:

- PAPILLARY THYROID CARCINOMA, TALL CELL VARIANT.

- ***Tumor size: 1.1cm.***
- ***Mitotic rate: 0-1 / 10 HPF***
- ***Capsular invasion: - Absent.***
- ***Vascular invasion: -Absent.***
- ***Microscopic extrathyroid tumor extension: -Absent.***
- ***Microscopic resection margins: -Negative.***
- ***Regional lymph nodes: Were not received.***

With The Best Regards: K.Shirneshan MD. A.P.C.P

دکتر کیوان شیرنشان

S98-3661

واحد آزمایشگاه پاتولوژی

سطح۶-آسیب شناسی تشریحی بررسی ظاهری بافت و ریزبینی (میکروسکوپی)شامل:حلق رزکسیون پارشیال/توتال بهمراه عقده های لنفاوی ناحیه

EXE.Time 1398/04/18 15:53 Result.Time 1398/05/05 17:29 Print.time

Macroscopic

نمونه ارسالی شامل دو ظرف می باشد:

۱-ظرف اول با برچسب لنف سنترال شامل یک قطعه بافت چربی به ابعاد ۰.۵×۱.۵×۱.۵ سانتیمتر می باشد.

۲-ظرف دوم با برچسب تیروئید شامل چندین قطعه بافت جداگانه جمعا به ابعاد ۱×۳×۷ سانتیمتر می باشد(به علت قطعه قطعه شدن تعیین مشخصات آناتومیکال امکان پذیر نمی باشد ، هم چنین شکل تیپیک لوب های تیروئید روئت نشد.)

در برشهای متوالی قطعات یک ندول کرمی رنگ به حداکثر قطر ۰.۸ سانتیمتر روئت شد.

Microscopic S98-3661

Procedure:**Total thyroidectomy with partial parathyroidectomy**

Specimen Integrity :**Not Intact (fragmented)**

Specimen Weight: **30 g**

Tumor Focality: **Unifocal**

Dominant Tumor:

- Laterality:**Right lobe**
- Tumor Size: **0.8 cm in greatest dimension**
- Histologic Type: **Papillary carcinoma (classic)**
- Margins: **Free**
- Capsular Invasion: **Not identified**
- Lymph-Vascular Invasion: **Not identified**

Pathologic Staging:

- Primary Tumor : **PT1a(Tumor < 1 cm in greatest dimension , limited to the thyroid)**
- Regional Lymph Nodes: **PNx (Regional lymph nodes not submitted)**
- Distant Metastasis: **PMx**

Staging Grouping:**Stage I**

Additional findings:

Recived secimen labeled as central lymph node contain a parathyroid gland and lymph node tissue was not seen.

C_code C۷۳,۹ M_code M۸۰۵۰/۳

از طرف دستیار : **دکتر رادفر شیوا**

Signature: [illegible]

[illegible]

Pat-9431

MACROSCOPIC DESCRIPTION:
The specimen consisted of thyroidectomy as one piece measured 6.5*2.5*1.5 cm. One ill defined solid mass gray to white (1.3 cm .in diameter).was seen in left lobe. Other parts of thyroid had heterogenous appearance.

DX: THYROIDECTOMY:

Left Lobe: -Tumor Type: Papillary Thyroid Carcinoma, Classic Type

-Tumor Size: 1.3 cm

-Tumor Mitosis: Low

-Tumor Necrosis: Absent

-Nuclear Atypia: Present, mild

-Tumor Dedifferentiation: Absent

-Encapsulation: Absent

-Capsular Invasion: Absent

-Closest Distance to Resected Margin: the mass is abutting the capsule

-Vascular invasion: Absent

-Perineurial invasion: Absent

-Extrathyroid Extension: Absent

-Surgical Margin: Free of tumor

-Tumor Multicentricity: Absent

-Additional Pathologic Findings: Lymphocytic thyroiditis of both lobes

Pathologic stage: T1b N0 M0: Stage I

.... Dr. Sh.Safabakhsh

آزمایشگاه تشخیص طبی و پاتولوژی دکتر شیرنشان
خیابان شمس آبادی-روبروی بیمارستان سینا-ساختمان فارابی-۰۳۱۳۲۳۴۵۰۳۰ p98_71465

MACROSCOPIC DESCRIPTION:

The specimen consists of thyroid, totally weigh 12g, left lobe measures 3x2x1cm, with brown color in cut section. The right lobe measures 4x2x1.5cm. In cut section in the right lobe in superior pole reveals a whitish area measures 0.8cm in diameter.

MICROSCOPIC DESCRIPTION:

In right lobe sections show foci of follicular variant of papillary carcinoma, with nuclear enlargement, ovarlapping, nuclear membrane irregularities and margination of chromatin to nuclear membrane are seen. Some abortive papillae are seen.

Dx: - TOTAL THYROIDECTOMY:

- FOLLICULAR VARIANT OF PAPILLARY THYROID CARCINOMA OF RIGHT LOBE.

- ***Tumor size: 0.8cm***
- ***Mitotic rate: 0-1 / 10 HPF***
- ***Capsular invasion: Present (Focal)< 4 Foci***
- ***Vascular invasion: Present (Focal)< 4 Foci***
- ***Microscopic extrathyroid tumor extension: - Absent.***
- ***Microscopic resection margins: - Negative.***
- ***Regional lymph nodes: were not received.***

With The Best Regards: K.Shirneshn M.D. A.P.C.P.

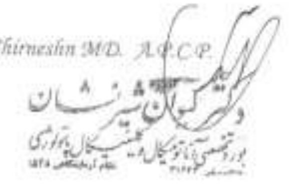

Supplement: Supplementary file 1 — Supplementary Information 1. [file 41598_2020_75495_MOESM1_ESM.pdf]
